# Dynamic graph cut based segmentation of mammogram

**DOI:** 10.1186/s40064-015-1180-7

**Published:** 2015-10-12

**Authors:** S. Pitchumani Angayarkanni, Nadira Banu Kamal, Ranjit Jeba Thangaiya

**Affiliations:** Department of Computer Science, Lady Doak College, Madurai, Tamil Nadu India; Department of M.C.A., TBAK College, Kilakarai, Ramnad, Tamil Nadu India; Department of M.C.A., Karunya University, Coimbatore, Tamil Nadu India

**Keywords:** Fuzzification, Graph cut, Otsu’s method and ROC

## Abstract

This work presents the dynamic graph cut based Otsu’s method to segment the masses in mammogram images. Major concern that threatens human life is cancer. Breast cancer is the most common type of disease among women in India and abroad. Breast cancer increases the mortality rate in India especially in women since it is considered to be the second largest form of disease which leads to death. Mammography is the best method for diagnosing early stage of cancer. The computer aided diagnosis lacks accuracy and it is time consuming. The main approach which makes the detection of cancerous masses accurate is segmentation process. This paper is a presentation of the dynamic graph cut based approach for effective segmentation of region of interest (ROI). The sensitivity, the specificity, the positive prediction value and the negative prediction value of the proposed algorithm are determined and compared with the existing algorithms. Both qualitative and quantitative methods are used to detect the accuracy of the proposed system. The sensitivity, the specificity, the positive prediction value and the negative prediction value of the proposed algorithm accounts to 98.88, 98.89, 93 and 97.5% which rates very high when compared to the existing algorithms.

## Introduction

The population based cancer registry evidently shows from the various statistics, that the incidence of breast cancer is rapidly rising, amounting to a significant percentage of all cancers in women. Breast cancer is the commonest cancer in urban areas in India and accounts for about 25–33% of all cancers in women. Over 50% of the breast cancer patients in India, being in stages 3 and 4 will definitely face the survival problem (Hassanien and Ali 2011). The survival rate can be increased only through early diagnosis. Image processing technique together with data mining is used for extraction and analysis of the ROI. Tumor can be classified into three categories normal, benign and malignant. A normal tumor is a mass of tissue which exists at the expense of healthy tissue. Malignant tumor has no distinct border. They tend to grow rapidly, increasing the pressure within the breast cells and can spread beyond the point from which they originate. Thus they grow faster than benign tumors and cause serious health problems if, left unnoticed. Benign tumors are composed of harmless cells and they have clearly defined borders. They can be completely removed and are unlikely to recur. MRI mammogram images taken after the appropriate segmentation of the tumor make classification of tumor into malignant, benign and normal a difficult task, due to complexity and variation in tumor tissue characteristics like its shape, size, grey level intensities and location. Effective segmentation techniques results in accurate classification of such cancerous masses.

## Data acquisition

A database of 1,528 mammograms, originating from the mammography image analysis society (MIAS), digital database for screening mammography, University of South Florida DDSM Resource, LLNL/UCSF database (Lawrence Livermore National Laboratories (LLNL), University of California at San Francisco) and Nijmegen digital mammogram database were used for the study.

## Methodology

### Image preprocessing and enhancement

The main objective behind the preprocessing step is to enlarge the intensity difference between objects and background. Preprocessing technique increases the optical inspection of an image. The proposed approach improves the image data by suppressing unwanted distortions and enhance the important image features. This will produce reliable representations of breast tissue structures. The fuzzy transformation function for computing the fuzzy plane value P is defined as follows:

- α = min
- β1 = (α + γ)/2
- β2 = (max + γ)/2
- γ = max/2

**Fig. 1:**
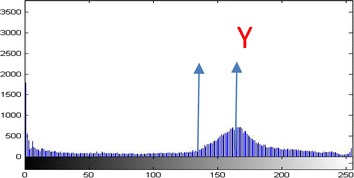
Histogram of the input image.

The histogram equalization of the gray levels in the original image can be characterized using five parameters:(α, β1, γ, β2, max). The aim is to decrease the gray levels below β1, and above β2. Intensity levels between β1 and γ, and β2 and γ are stretched in opposite directions towards the mean γ (Fig. 1).

*Procedure:*

Step 1: Fuzzification:

The following fuzzy rules are used for contrast enhancement:

Rule-1:

If α ≤ u_i_ < β1 then P = 2 ((u_i_ − α)/(γ − α))^2^

Rule-2:

If β1 ≤ u_i_ < γ then P = 1 − 2 ((u_i_ − γ)/(γ − α))^2^

Rule-3:

If γ ≤ u_i_ < β2 then P = 1 − 2((u_i_ − γ)/(max − γ))^2^

Rule-4:

If β2 ≤ u_i_ < max then P = 2 ((u_i_ − γ)/(max − γ))^2^

where u_i_ = f(x,y) is the ith pixel intensity

Step 2: Fuzzy Modification

Step 3: Defuzzification

The quality of the preprocessed image is to be checked with the following parameters like peak signal to noise ratio (PSNR), noise standard deviation (NSD), mean square error (MSE), equivalent number of looks (ENL).

### Image segmentation and ROI extraction

The region of interest i.e. the tumor region is segmented using the Graph cut method. The main purpose of using this method for segmentation is that it segments the mammogram into different mammographic densities. It is useful for risk assessment and quantitative evaluation of density changes. Apart from the above advantage it produces the contour (closed region) or a convex hull which is used for analyzing the morphological and novel features of the segmented region. The above technique results in efficient formulation of attributes which helps in classification of the ROI into benign, malignant or normal. Graph cuts have been used in recent years for interactive image segmentation (Hassanien and Badr 2003). The core ideology of graph cuts is to map an image onto a network graph, and construct an energy function on the labeling, and then do energy minimization with dynamic optimization techniques. This study proposes a new segmentation method using iterated graph cuts based on multi-scale smoothing. The multi-scale method can segment mammographic images with a stepwise process from global to local segmentation by iterating graph cuts. The modified graph cut approach used by K. Santle Camilus (Hassanien and Badr 2003) is implemented in this project.

Steps involved in graph cut segmentation are:

1. Form a graph
2. Sort the graph edges
3. Merging regions based on threshold

From the mammogram image a graph G = (V, E) is constructed such that V represents the pixel values of the 3 × 3 image and E represents the edges defined between the neighboring pixels. The weight of any edge W(Vi, Vj) is a measure of dissimilarity between the pixels Vi and Vj. The weight for an edge is measured by means of considering the Euclidian distance between the two pixels Vi and Vj (Ertas et al. 2001; Shah et al. 2011; Masek et al. 2001; Thamaraichelvi and Yamuna 2013; Jayadevappa et al. 2009; Benfield et al. 2007; Elnakib et al. 2011). It is represented by the equation

1 $$ {\text{W}}\left( {{\text{Vi}},{\text{Vj}}} \right) = \sqrt {(\varvec{xi} - \varvec{xj})^{2} + (\varvec{yi} - \varvec{yj})^{ 2} } $$

$$ {\text{Vi}} = \left( {{\text{xi}},{\text{yi}}} \right)\quad {\text{Vj}} = \left( {{\text{xj}},{\text{yj}}} \right) $$

*Procedure:*

1. Sort the edges in ascending order of their weights such that W(e_1_) ≤ W(e_2_).
2. Pick one edge e_i_ in the sorted order from e_i_ to e_n_ where e_i_ is between two groups of pixels which determines whether to merge the two groups of pixel to form a single group or not. Each vertex is considered as a group. The two groups which satisfies the merge criteria are merged together. The different groups of pixels representing different regions or objects are obtained.
3. *Determining the merge criteria*: When the pixels of a group have intensity values similar to the pixels of the other group, then intuitively the calculated IRM between these groups should be small. The expected smaller value of the IRM to merge these two regions is tested by comparing it with the dynamic threshold. Hence, the merge criterion, to merge the two regions, *R*_1_ and *R*_2_, is defined as:
  $$ {\text{Merge}}\left( {{\text{R}}_{ 1} ,{\text{R}}_{ 2} } \right),\quad {\text{if IRM}}\left( {{\text{R}}_{ 1} ,{\text{R}}_{ 2} } \right) \le DT({\text{R}}1,R2) $$

**Fig. 2:**
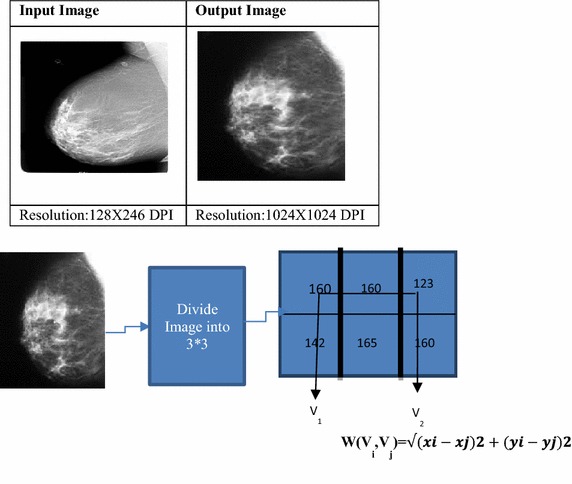
Weight calculation for the 3 × 3 matrix.

**Fig. 3:**
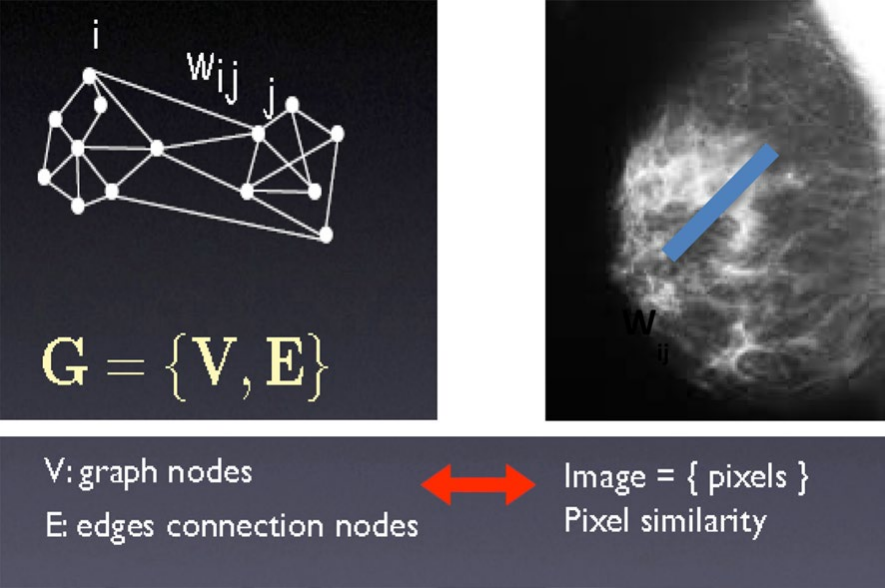
Graph cut approach.

**Fig. 4:**
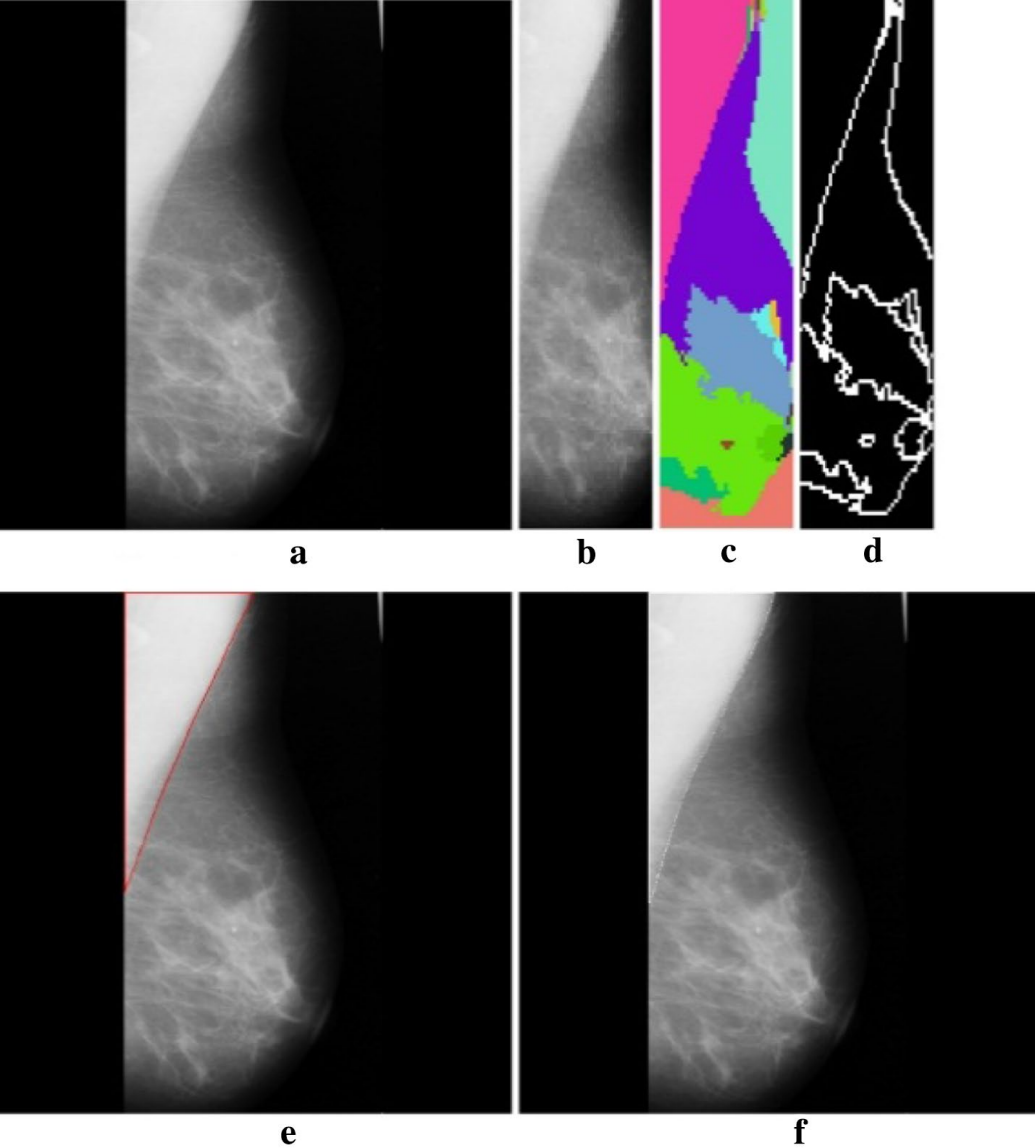
**a** Input image, **b** ROI, **c** segmented boundaries, **d** edge, **e** pectoral muscle identification indicated by *red color*, **f** ground truth value represented by *white*.

**Fig. 5:**
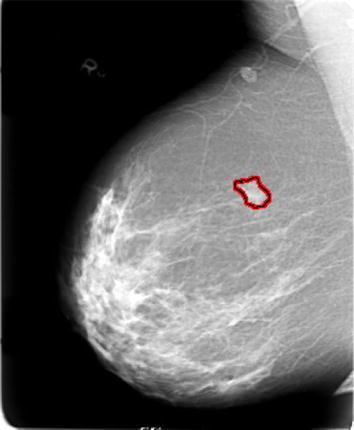
Segmented image.

Figure 2 specifies the weighted calculation applied to the input image. Figure 3 shows how graph cut method is applied on a 3 × 3 image. Figure 4 shows the stage by stage output of the proposed method and the segmented region is shown in Fig. 5.

## Performance analysis

Performance measure of the proposed mathematical approach at each stage was estimated.

### Preprocessing

Tabulation in Table 1 clearly shows a high PSNR value which shows that the image is highly enhanced (Camilus et al. 2010).

**Table 1:** PSNR tabulation

PSNR	RMS	H	$\gamma$	MSE	Nature of filter
87.65	2.97	0.2111	0.0086	8.83	FHQ

### Segmentation

The Table 2 below depicts the interpretation between the two approaches using the quantitative measures to determine the overall classification accuracy (Zhang et al. 2012; Annamalai et al. 2009; Ramaswamy and Rose 2009; Peng et al. 2010; Artan et al. 2012).

**Table 2:** Segmentation technique comparision

Parameters	Hassanien method	Proposed method
Target to background contrast measure based on standard deviation	0.71	0.83
Target to background contrast measure based on entropy	0.76	0.90
Index of fuzziness	0.2892	0.010
Fuzzy entropy	0.1056	−0.001
PSNR	86.75	90.88

### Segmentation accuracy

Segmentation accuracy is depicted in Table 3.

**Table 3:** Segmentation accuracy metrics

Specificity	95.5%
Sensitivity	97.3%
Positive prediction value	89%
Accuracy	98.9%
Area under curve	0.98
Negative prediction value	98%

### Computational efficiency

Table 4 clearly depicts the computational efficiency of the proposed method is efficient compared to the other existing technique.

**Table 4:** Computational efficiency of the proposed method

Methods	References	System specification	Computational time based on implementation
Rough set approach	Hassanien and Ali (2011)	Intel Pentium^®^ CPU B950 Processor 2 GB RAM 32-bit OS Windows 7	2′19″
Mathematical Morphological	Bojar and Nieniewski (2008)		2′50″
Shape and texture feature	Zakeri et al. (2012)		8′21″
Shape, edge-sharpness, and texture features	Mu et al. (2008)		0′45″
Proposed method	Angayarkanni et al. (2002)		0′03″

### Metrics for evaluating the segmentation technique includes

The region-based criteria mutually compare the machine segmented regions with the correct ground truth regions.

Let A(I, J) denote the machine segmented region and B(I, J) denotes the ground truth region then the region overlap acceptance is controlled by the threshold k = 0.75 then

*Region overlap* Local refinement error

$$ {\text{E}}\left( {{\text{A}},{\text{B}},{\text{k}}} \right) = {{\left| {{\text{R}}\left( {{\text{A}},{\text{k}}} \right)/{\text{R}}\left( {{\text{B}},{\text{k}}} \right)} \right|} \mathord{\left/ {\vphantom {{\left| {{\text{R}}\left( {{\text{A}},{\text{k}}} \right)/{\text{R}}\left( {{\text{B}},{\text{k}}} \right)} \right|} {{\text{R}}\left( {{\text{A}},{\text{k}}} \right)}}} \right. \kern-0pt} {{\text{R}}\left( {{\text{A}},{\text{k}}} \right)}} $$

*Edgel matching* Overlay the original with segmented image and compute correspondence via min-cost assignment on bipartite graph.

**Fig. 6:**
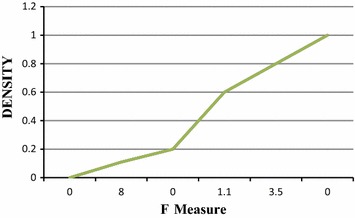
F-measure.

The F-measure value is shown in Fig. 6.

## Conclusions

The proposed mathematical approach yields a high level of accuracy within a minimum period of time that confirms the efficiency of the algorithm. The GUI based CAD system was developed using Scilab and R2. The segmentation speed accounts to 6 ms using graph cut based Otsu’s thresholding. The main goal of classifying the tumors into benign, malignant and normal is achieved with a great accuracy compared to other techniques because of the implementation of the accurate segmentation technique employed. The proposed technique is computationally efficient as specified in the tabulation above. Further the complexity of the algorithm in asymptotic sense is equivalent to o(log n).
